# Studies on Immunogenicity and Antigenicity of Baculovirus-Expressed Binding Region of *Plasmodium falciparum* EBA-140 Merozoite Ligand

**DOI:** 10.1007/s00005-015-0367-5

**Published:** 2015-10-06

**Authors:** Agata Zerka, Joanna Rydzak, Anna Lass, Beata Szostakowska, Wacław Nahorski, Agnieszka Wroczyńska, Przemyslaw Myjak, Hubert Krotkiewski, Ewa Jaskiewicz

**Affiliations:** Ludwik Hirszfeld Institute of Immunology and Experimental Therapy, Polish Academy of Sciences, Wrocław, Poland; Department of Tropical Parasitology, Institute of Maritime and Tropical Medicine in Gdynia, Medical University of Gdańsk, Gdańsk, Poland; Department of Tropical and Parasitic Diseases, Institute of Maritime and Tropical Medicine in Gdynia, Medical University of Gdańsk, Gdańsk, Poland; Department of Molecular Biology, Faculty of Biological Sciences, University of Zielona Góra, Zielona Gora, Poland

**Keywords:** *Plasmodium falciparum*, Recombinant binding region of EBA-140 ligand, Region II baculovirus expression, Immunogenicity, Antigenicity, Natural anti-EBA-140 IgG antibodies

## Abstract

The erythrocyte binding ligand 140 (EBA-140) is a member of the *Plasmodium falciparum* erythrocyte binding antigens (EBA) family, which are considered as prospective candidates for malaria vaccine development. EBA proteins were identified as important targets for naturally acquired inhibitory antibodies. Natural antibody response against EBA-140 ligand was found in individuals living in malaria-endemic areas. The EBA-140 ligand is a paralogue of the well-characterized *P. falciparum* EBA-175 protein. They both share homology of domain structure, including the binding region (Region II), which consists of two homologous F1 and F2 domains and is responsible for ligand–erythrocyte receptor interaction during merozoite invasion. It was shown that the erythrocyte receptor for EBA-140 ligand is glycophorin C-a minor human erythrocyte sialoglycoprotein. In studies on the immunogenicity of *P. falciparum* EBA ligands, the recombinant proteins are of great importance. In this report, we have demonstrated that the recombinant baculovirus-obtained EBA-140 Region II is immunogenic and antigenic. It can raise specific antibodies in rabbits, and it is recognized by natural antibodies present in sera of patients with malaria, and thus, it may be considered for inclusion in multicomponent blood-stage vaccines.

## Introduction

Malaria due to *Plasmodium falciparum* is one of the most significant causes of morbidity and mortality globally accounting for above a half million deaths each year (Miller et al. 2013; WHO 2014). It is also the most frequently imported acute, life-threatening tropical disease in international travelers (Lüthi and Schlagenhauf 2015). *P. falciparum* merozoite antigens which play a pivotal role in the recognition and invasion of the parasite into human red blood cells are likely targets of protective immune responses (Ahmed Ismail et al. 2014; Crompton et al. 2014; Fowkes et al. 2010; Osier et al. 2008; Richards et al. 2013). It is anticipated that immunization with a combination of merozoite proteins could elicit antibodies which might block erythrocyte invasion (Healer et al. 2013; Pandey et al. 2013; Richards et al. 2013).

Erythrocyte invasion by *Plasmodium* spp. is a complex process (Bei and Duraisingh 2012; Cowman et al. 2012; Gaur and Chitnis 2011). Several merozoite-stage proteins that have a role during invasion have been extensively studied, including merozoite surface proteins (MSP), AMA-1 antigen, erythrocyte-binding-like ligands (EBL: EBA-175, EBA-181, and EBA-140) and reticulocyte-binding-like ligands (RBL or PfRh: PfRh1, PfRh2a, PfRh2b, PfRh4, and PfRh5) (Jaskiewicz et al. 2010; Malpede and Tolia 2014; Tham et al. 2012). However, it still remains unclear which merozoite invasion ligands might be the most important targets of naturally acquired clinical immunity. Erythrocyte-binding antigens (EBA) (Adams et al. 2001; Tham et al. 2012) are particularly promising as the targets of protective immunity, but there are limited data examining their potential importance. Indeed, all the three EBA ligands were identified as important targets of naturally acquired inhibitory antibodies (Persson et al. 2013; Richards et al. 2010, 2013). Natural antibody response against EBA-140 ligand was found in individuals living in malaria-endemic areas (Ford et al. 2007; Richards et al. 2010, 2013; Stanisic et al. 2015) and in low-transmission malaria regions (Villasis et al. 2012).

The EBA-140 ligand (Gilberger et al. 2003; Narum et al. 2002; Thompson et al. 2001), a paralog of the well-characterized *P. falciparum* EBA-175 protein (Tolia et al. 2005) was shown to bind to glycophorin C (GPC) (Jaskiewicz 2007; Lobo et al. 2003; Maier et al. 2003; Rydzak et al. 2013), a minor erythrocyte membrane sialoglycoprotein (Cartron et al. 1993; Jaskiewicz et al. 2002a; Lisowska 1988), mediating a distinct invasion pathway into human erythrocytes. The EBA-140 ligand binds to erythrocytes in a sialic acid-dependent manner, and it was proposed that the receptor for the EBA-140 ligand might be a cluster of N- and O-linked sialylated glycans on the GPC molecule (Jiang et al. 2009; Lin et al. 2012; Mayer et al. 2006). Recently, the crystal structure of the recombinant EBA-140 erythrocyte-binding region (Region II), obtained in bacteria, in a complex with a glycan-containing sialic acid has been characterized, and the role of individual glycan-contacted amino acid residues in specific sialic acid interactions was revealed (Lin et al. 2012; Malpede et al. 2013). Since the EBA-140 ligand failed to bind the natural deletion variant of GPC Gerbich-type (Jiang et al. 2009; Maier et al. 2003, 2009; Mayer et al. 2002, 2006; Rydzak et al. 2015), which lacks amino acid (aa) residues 36–63 (Jaskiewicz et al. 2002b; Kusnierz-Alejska et al. 1990; Walker and Reid 2010), it was suggested that this GPC region and GPC oligosaccharide chains play a crucial role in the EBA-140 ligand binding (Ashline et al. 2015; Maier et al. 2003, 2006).

The major limitation in the studies on *P. falciparum* EBA ligands is their expression and purification as soluble and properly folded recombinant proteins in sufficient amounts. The soluble, recombinant EBA-140 Region II and its F2 domain was obtained in bacteria (Lin et al. 2012; Rydzak et al. 2012), *P. pastoris* (Ford et al. 2007; Richards et al. 2010) or in the baculovirus expression system (Kobayashi et al. 2010; Rydzak et al. 2015). Region II of the EBA-140 antigen was also expressed on the surface of Chinese hamster ovary (CHO-K1) cells (Jiang et al. 2009), COS7 cells (Martin et al. 2005; Mayer et al. 2002) and HEK-293T cells (Malpede et al. 2013), as a functional but insoluble, membrane-bound recombinant protein.

Previously, we have reported baculovirus expression of EBA-140 Region II and characterization of its binding properties to GPC (Ashline et al. 2015; Rydzak et al. 2012, 2015). In this report, we have demonstrated that the recombinant baculovirus-obtained EBA-140 Region II is immunogenic and antigenic, since it can raise specific antibodies in rabbits and it is recognized by natural antibodies present in sera of patients with malaria. These results suggest that the functionally active recombinant Region II of EBA-140 ligand may be considered for inclusion in multicomponent asexual blood-stage vaccines.

## Materials and Methods

### Recombinant Proteins

Recombinant baculovirus containing the EBA-140 Region II cDNA sequence coding for aa residues 141–756 with 6×His and c-myc tags at its C-terminus, was obtained by GeneScript (Hong Kong). The high titer (2 × 10^8^ pfu/ml) virus was inoculated into SF9 cells cultured in one liter of CCM3 serum-free medium at MOI 5 (multiplicity of infection). The soluble, recombinant Region II secreted into the medium was purified by Ni–NTA affinity chromatography, as described previously (Rydzak et al. 2015).

Recombinant F2 domain of EBA-140 antigen was expressed in *Escherichia coli* Rosetta-gami as the fusion protein with maltose binding protein at the N-terminal end and with c-myc, 6×His tags at the C-terminus. The recombinant F2 domain fragment (aa residues 561–756) was purified from bacterial lysate by Ni–NTA affinity chromatography, as described previously (Rydzak et al. 2012).

### SDS-PAGE

The proteins were separated by electrophoresis in the presence of SDS using 10 % polyacrylamide gel under denaturing conditions, according to Laemmli method (Laemmli 1970). The PageRuler Prestained Protein Ladder (Fermentas, Lithuania) was used as a molecular weight protein marker.

### Western Blotting

Recombinant proteins fractionated by SDS-PAGE were transferred to nitrocellulose membrane (Schleicher & Schuel, Germany) according to the method of Towbin et al. (1979) and detected with mouse monoclonal antibody (MoAb) directed against c-myc epitope (clone 9E10, ATCC) or with rabbit polyclonal serum.

### Rabbit and Human Sera

#### Rabbit Sera

Sera were obtained from rabbits immunized with 50 μg of baculovirus-expressed EBA-140 Region II in monophosphoryl lipid A (MPL) adjuvant as described previously (Rydzak et al. 2012).

#### Human Sera

Human serum samples used for enzyme-linked-immunosorbent assay (ELISA) were collected from patients of the Institute of Maritime and Tropical Medicine in Gdynia (Poland) (Goljan et al. 2003). Twenty-five samples were obtained from 19 adults with imported malaria, diagnosed after return to Poland from travel to tropical regions (group 1). In six patients, blood was sampled twice for the measurements: in the acute stage of infection and in the recovery period (1–3 months after the disease symptoms resolution; Table 1). Sera were also collected from 11 patients (missionaries) with the history of previous *Plasmodium* spp. infection and positive result of malaria serological examination, but without clinical illness at the time of sampling (group 2). Nine control samples were obtained from the Institute employees (group 3). Serum samples had been kept in frozen condition at the temperature of −20 °C until the assays were performed.

Malaria was confirmed with microscopic examination (standard Giemsa stained thick and thin blood smears), indirect immunofluorescence assay (IFA) (Myjak et al. 1993), and PCR (Myjak et al. 2002). Result of IFA assay is shown in the form of titer—dilution of the serum at which the result is positive. In the IFA test, whole parasite obtained from culture of *P. falciparum* serves as antigen.

Participants provided their written informed consent for routine diagnostic procedures, which were used to obtain the study material. The study was approved by the Ethics Committee of Medical University in Gdansk, Poland (No. NKEBN/46/2005, the approval was given to A.W.).

### ELISA

Microtitre plates (Nunc, Fisher Scientific) were coated with the recombinant Region II or its truncated F2 domain (0.5 μg/well) in carbonate buffer pH 9.6 at 4 °C overnight, and then blocked for 2 h with 5 % milk powder solution in TBST (50 mM Tris–HCl, 150 mM NaCl, pH 7.4 containing 0.05 % Tween 20). Serial dilutions (2-fold) of rabbit serum starting with 200-fold dilution or MoAb anti-myc starting with 10-fold dilution was incubated for 1 h at room temperature in TBST. After washing with TBST, the binding was determined with goat anti-rabbit Ig antibody or rabbit anti-mouse antibody conjugated with alkaline phosphatase (DakoCytomation, Denmark). Alternatively, only with Region II-coated plates, the human sera from malaria patients were used in 200-fold dilution, and the reaction was determined with anti-human IgG antibody conjugated with alkaline phosphatase (Sigma, USA). The absorbance at 405 nm was read using EnSpire Multilabel Reader (Perkin Elmer, USA). Non-immune rabbit serum or sera from healthy donors, respectively, were used as the negative control. All data are mean values of experiments performed in triplicate. Blank test (buffer instead of serum) optical density (OD) value was subtracted from each absorbance. Results above the mean OD value of malaria-negative controls (0.462) plus 2 SD for each response (=0.684) were considered seropositive.

### Flow Cytometry Analysis

The binding of the recombinant Region II (0.5, 1.0, 3.0 µg) was assayed with 3 × 10^5^ human red blood cells (RBCs) in 100 µl of phosphate-buffered saline (PBS), pH 7.4 for 2 h at 4 °C. After three washings with PBS, RBCs were incubated for 1 h at 4 °C with rabbit serum (1:200) for 1 h at 4 °C. Alternatively, an inhibition assay was performed with dilutions of rabbit serum (1:500, 1:1000, 1:2000) in PBS incubated with 0.5 µg of Region II for 2 h at room temperature, and then added to RBC for 30-min incubation at 4 °C. After three washings with PBS, RBCs were finally incubated for 1 h at 4 °C with FITC-conjugated goat anti-rabbit Ig antibody (DakoCytomation, Denmark). Erythrocytes were analyzed for fluorescence intensity using flow cytometry (FACS Calibur, BD Biosciences, USA). Data were analyzed using Flowing Software 2.5.1. Mean fluorescence intensity was calculated after subtraction of a negative control value (0 µg Region II).

## Results

### Immunoreactivity of Baculovirus-Expressed EBA-140 Region II

**Fig. 1:**
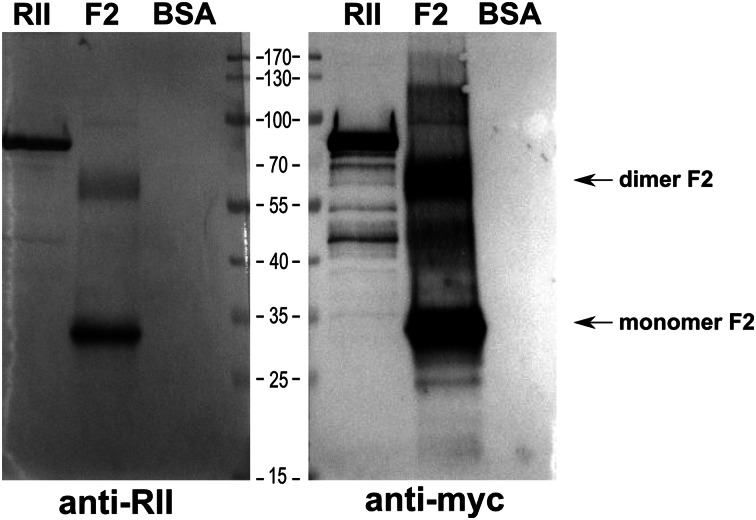
Recognition of the recombinant EBA-140 Region II (RII) and its F2 domain fragment by MoAb (anti-myc, clone 9E10) and polyclonal rabbit antibodies (anti-RII) in Western blotting; bovine serum albumin (BSA), negative control

Recombinant Region II of EBA-140 ligand and its F2 domain fragment were expressed in Sf9 insect cells or bacteria (*E. coli,Rosetta gami*) respectively, and their binding specificities were characterized (Rydzak et al. 2012, 2015). The recombinant proteins of predicted molecular masses: Region II (~75 kDa) and F2 domain fragment (~26 kDa), tagged with c-myc epitope at the C-terminus were detected in immunoblotting using anti-myc MoAb (Fig. 1).

It was shown that baculovirus-obtained Region II and its truncated form-F2 domain fragment obtained in bacteria are recognized by polyclonal rabbit antibodies raised against the whole EBA Region II in MPL adjuvant (Rydzak et al. 2012) (Fig. 1).

**Fig. 2:**
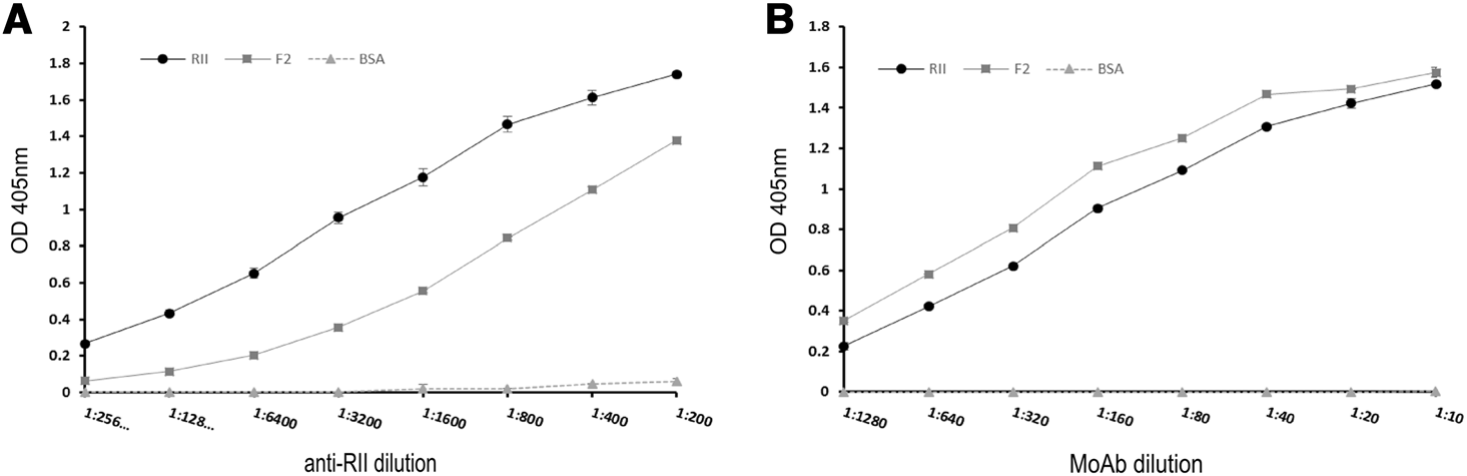
Binding of rabbit antibodies to the recombinant EBA-140 Region II and F2 domain fragment in ELISA (**a**). MoAb anti-myc (clone 9E10) was used as a positive binding control (**b**); BSA, negative control

An ELISA assay was also performed to evaluate immunoreactivity of the recombinant Region II obtained in insect cells and its bacterial F2 domain fragment with polyclonal antibodies obtained from a rabbit. As a binding control, monoclonal anti-myc antibody was used (Fig. 2).

These results indicate that the recombinant baculovirus-expressed Region II is immunogenic and can raise specific antibodies which recognize Region II and its F2 domain fragment obtained in bacteria, as well. However, the recognition of F2 domain’s truncated form in quantitative ELISA assay is weaker than that in the whole Region II, perhaps due to its shorter polypeptide consisting of 194 aa residues instead of 614 of Region II.

### Inhibition of EBA-140 Region II Binding to Erythrocytes

**Fig. 3:**
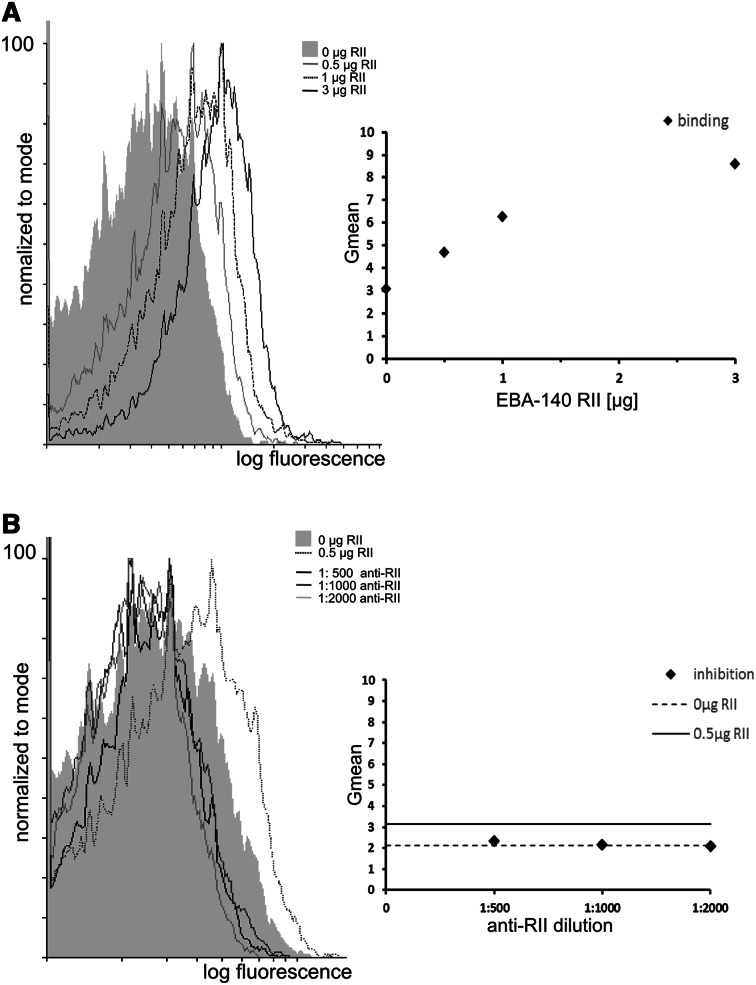
**a** Binding of the recombinant EBA-140 Region II (RII) to human erythrocytes mesured in flow cytometry; **b** Inhibition of the erythrocyte binding of the recombinant EBA-140 Region II by rabbit antibodies (anti-RII)

Analysis of the erythrocyte binding by the recombinant EBA-140 Region II was performed by cytofluorymetry in a dose-dependent manner (Fig. 3a). Polyclonal rabbit antibodies were tested for an inhibition of the Region II binding to erythrocytes (Fig. 3b).

It was shown that the EBA-140 Region II binding to erythrocytes can be efficiently blocked by rabbit antibodies in the following dilutions: 1:500, 1:1000, and 1:2000.

### Recognition of Recombinant EBA-140 Region II by Human Antibodies

To determine whether the baculovirus-expressed recombinant EBA-140 Region II would react with natural antibodies present in human sera, an ELISA assay was used. Sera were obtained from the returned travelers with acute malaria imported to Poland (group 1) or patients with the history of previous malaria during their stay in tropical regions (group 2).

Reactivity with EBA-140 recombinant antigen was observed not only with regard to *P. falciparum* malaria cases (12/19) but also in sera from patients infected with *P. vivax* (3/4 sera) and *P. malariae* (1/1), while result for one *P. ovale* was negative (Table 1). In four of six patients, who were examined twice at 1–3 months intervals, correlation between IFA titer and extinction (OD) obtained with recombinant antigen was observed.

**Table 1:** Laboratory results obtained with human sera from the acute malaria patients, patients in the recovery period, and the control group

Group	Sample no.	Interval in days	Results				
			Microscopic	PCR	IFA	EBA-140 Region II	
						OD	Results
1	1 a 2 a*	0 62	*P. falciparum*	*P. falciparum*	1:160 1: 1280	0.915 1.962	Positive Positive
	3 b 4 b*	0 46	*P. falciparum*	Not done	1:20 1:320	0.833 1.333	Positive Positive
	5 c 6 c*	0 98	*Plasmodium* sp.	*P. falciparum* + *P. vivax*	1:640 1:80	0.794 0.482	Positive Negative
	7 d 8 d*	0 57	*P. falciparum*	*P. falciparum*	1:640 1:2560	0.453 0.483	Negative Negative
	9 e 10 e*	0 51	*Plasmodium* sp.	*P.falciparum*	1:80 1:1280	0.816 0.568	Positive Negative
	11 f 12 f*	0 37	*P. falciparum*	Not done	1:2560 1:320	1.067 0.547	Positive Negative
	13	0	*P. falciparum*	Not done	1:20	0.837	Positive
	14	0	*Plasmodium* sp.	*P. falciparum*	1:2560	1.143	Positive
	15	0	Negative	*P. falciparum*	1:640	1.205	Positive
	16	0	*Plasmodium* sp.	*P. falciparum*	1:80	1.143	Positive
	17	0	*P. falciparum*	Not done	1:1280	1.497	Positive
	18	0	*P. falciparum*	Not done	Not done	0.477	Negative
	19	0	*P. falciparum*	Not done	Not done	0.431	Negative
	20	0	*P. ovale*	Not done	1:160	0.408	Negative
	21	0	*P. vivax*	*P. vivax*	Negative	0.97	Positive
	22	0	Negative	*P. vivax*	1:20	0.647	Negative
	23	0	*Plasmodium sp.*	*P. vivax*	1:160	1.353	Positive
	24	0	*P. vivax*	Not done	1:80	1.192	Positive
	25	0	*P.malariae*	Not done	1:20	1.159	Positive
2	26–36	Period not known	Negative	Not done	1:160–1:2560	0.528–3.042	7 positive 4 negative
3	37–45		Negative	Not done	Negative	0.280–0.647	Negative

* Samples were obtained from the same patient (a, b, c, d, e, f)

**Fig. 4:**
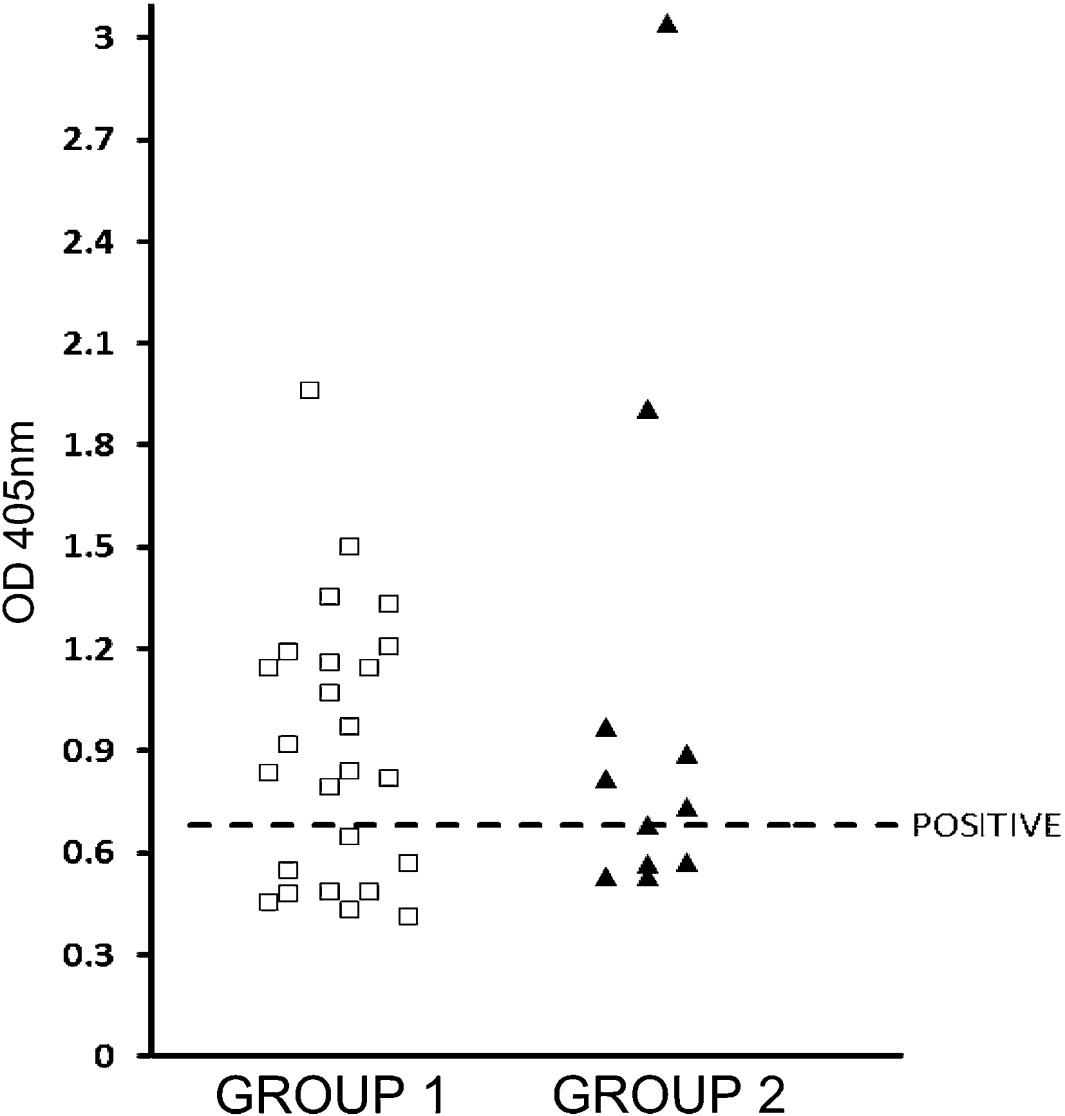
Reactivity of human sera from persons with acute malaria (group 1, *n* = 25) and with the history of *Plasmodium* infection confirmed by serological examination (group 2, *n* = 11) with the recombinant EBA-140 Region II in ELISA

A pattern of reactivity shown in Fig. 4 indicates that 64 % of all samples (23 of 36) were seropositive with EBA-140 recombinant antigen. The level of serum antibodies to EBA-140 in persons with the past history of malaria (7/11 = 63.6 %, group 2) seems to be similar to values seen in the patients in the acute stage (16/25 = 64 %, group 1) of symptomatic *Plasmodium* infection.

## Discussion

The immunological characterization of recombinant vaccine candidate antigens is important in determining their identity to native *P. falciparum* proteins. We sought to determine whether the recombinant baculovirus-obtained Region II and its truncated F2 domain were both antigenic and immunogenic.

A pattern of reactivity shown in Fig. 4 indicates that 64 % of all samples were seropositive, indicating that EBA-140 ligand mediating one of invasion pathways is recognized, in considerable amount, by natural human antibodies. High numbers of seropositive samples were found in patients infected with *P. falciparum* as well as in patients infected with *P. vivax* and *P. malariae*. Thus, EBA-140 ligand seems to be a target of immunity in human malaria caused not only by *P. falciparum*, but also by other *Plasmodium* species.

*Plasmodium* cross-species immunity was mostly reported for whole live-attenuated sporozoites (Douradinha et al. 2008; Good et al. 2013; Mauduit et al. 2009); however, there are only a few reports regarding the use of recombinant *Plasmodium* proteins. Using a murine model, it was demonstrated recently that antibodies generated in rabbits or mice against the recombinant *P. vivax* circumsporozoite protein (CSP) recognize CSP on the surfaces of *P. falciparum* and *P. berghei* sporozoites as well, but the titers were very low (Yadava et al. 2012). Moreover, the capacities of the recombinant *P. falciparum* cell-traversal protein for ookinetes and sporozoites (CelTOS) to induce sterile protection in mice against a challenge with *P. berghei* sporozoites were achieved (Bergmann-Leitner et al. 2010). Cross-species interactions were also observed with asexual blood stages, but these interactions are even less understood (Douradinha et al. 2008). For example, it was found that natural exposure to *Plasmodium* species induces anti-MSP5 IgG responses which cross-react with *P. falciparum* and *P. vivax*, albeit infrequently (Woodberry et al. 2008).

Because of the conserved nature of EBA proteins concerning Duffy-binding-like (DBL) domains structure of all *Plasmodia* species, induction of antibodies specific for these domains could result in cross-reactive immune response. Cross-reactivity of human natural antibodies specific for anti-EBA-140 ligand, observed in the study suggests that this protein might be considered as useful antimalarial vaccine candidate, although the antibody titers were mostly low.

Two major conclusions can be drawn from this study. The first and the most important is that the recombinant baculovirus-obtained Region II is expressed and folded in such a way that it can be recognized by immune sera from persons with acute malaria imported to Poland and in returned travelers, exposed previously to *Plasmodium* spp. in endemic areas of different locations. Recently, an extensive evaluation of the antigenic properties of recombinant EBA-140 ligand was presented. In the report examining the humoral response to the recombinant EBA-140 invasion ligand obtained in yeast, considerable IgG1 and IgG3 antibodies were detected in Cameroonian population (Ford et al. 2007). High levels of IgG against three EBA ligands, including EBA-140 were shown to be strongly associated with a protection from symptomatic malaria and high-density parasitemia (among a cohort of 206 Papua New Guinea children) IgG seropositivity to recombinant EBA-140 Region II obtained in bacteria was very high (85.4 %) (Richards et al. 2010). Based on these studies, the EBA-140 ligand appears as an important target of acquired protective immunity and the recombinant proteins are a useful tool to study its immunogenic properties.

Second, we demonstrated that baculovirus-expressed recombinant Region II, when used as an immunogen, elicited in rabbits antibodies that recognize the whole Region II and its truncated domain F2 in Western blotting. We employed two immunological assays to evaluate the immune response: ELISA assay to measure antibody level and FACS analysis to test the inhibitory capacity of rabbit immune serum to block Region II ligand–receptor interaction on human erythrocytes. We were able to show that obtained rabbit antibodies were efficient in the inhibition of the binding of the recombinant EBA-140 Region II to erythrocytes in high dilutions. We anticipate that these assays can facilitate the analysis of the immunogenicity of EBA-140 as a vaccine candidate.
